# Supplementary material for: Association of combination statin and antihypertensive therapy with reduced Alzheimer’s disease and related dementia risk
Source: PLoS One. 2020 Mar 4;15(3):e0229541. doi: 10.1371/journal.pone.0229541 (PMC7055882; doi:10.1371/journal.pone.0229541)
Supplement: S1 Appendix — (DOCX) [file pone.0229541.s001.docx]

**S1 Appendix: Summary of ICD-9 codes used to define various diagnoses and symptoms**

AD 331.0

ADRD Alzheimer's Disease and Related Disorders or Senile Dementia 3 years DX 331.0, 331.11, 331.19, 331.2, 331.7, 290.0, 290.10, 290.11, 290.12, 290.13, 290.20, 290.21, 290.3, 290.40, 290.41, 290.42, 290.43, 294.0, 294.10, 294.11, 294.20, 294.21, 294.8, 797 (any DX on the claim)
