# Supplementary material for: Association of combination statin and antihypertensive therapy with reduced Alzheimer’s disease and related dementia risk
Source: PLoS One. 2020 Mar 4;15(3):e0229541. doi: 10.1371/journal.pone.0229541 (PMC7055882; doi:10.1371/journal.pone.0229541)
Supplement: S1 Table — Logistic regression results for AD incidence in sample of 2009–2014 Medicare person-years with 90 possession days and 2 claims of both an AHT and a statin in both years t-1 and t-2. AHTs are antihypertensive (AHT) prescription drugs (angiotensin converting enzyme inhibitors (ACEIs), angiotensin-II receptor blockers (ARBs), beta-blockers, calcium channel blockers, loop diuretics, and thiazide diuretics), and statins are atorvastatin, pravastatin, rosuvastatin, and simvastatin. Sample restricted to person-years with 3 years fee-for-service, 3 years Part D, age 67+, no deaths in the reference year (year t), no prior AD diagnoses, and no prior use of acetylcholinesterase inhibitors (AChEIs) or memantine. Controls are age, age squared, sex, education, income quartiles, statin use (t-1), years since hypertension and hyperlipidemic diagnoses, HCC comorbidity index, number of physician visits, and indicators for past diagnoses of diabetes, atrial fibrillation, acute myocardial infarction, and stroke. Standard errors are clustered at the county level. (DOCX) [file pone.0229541.s002.docx]

| **S2 Table: Adjusted odds ratios of AD incidence associated with use of statin-AHT combinations, relative to users of other statin-AHT combinations** | | | | | | | | |
| --- | --- | --- | --- | --- | --- | --- | --- | --- |
| **Statin** | **AHT** |  | **All** | **Female** | **Male** | **White** | **Black** | **Hispanic** |
| **Ator** | **ACEI** | OR | 1.025 | 1.068 | 0.936 | 1.039 | 0.937 | 0.957 |
|  |  | CI | (0.976-1.077) | (1.006-1.134) | (0.859-1.021) | (0.983-1.098) | (0.784-1.120) | (0.799-1.145) |
|  |  | p | 0.324 | 0.032 | 0.135 | 0.176 | 0.476 | 0.630 |
|  |  |  |  |  |  |  |  |  |
| **Sim** | **ACEI** | OR | 1.010 | 1.010 | 1.007 | 1.009 | 1.011 | 0.985 |
|  |  | CI | (0.972-1.049) | (0.964-1.058) | (0.941-1.077) | (0.967-1.054) | (0.894-1.143) | (0.868-1.118) |
|  |  | p | 0.610 | 0.673 | 0.848 | 0.678 | 0.859 | 0.813 |
|  |  |  |  |  |  |  |  |  |
| **Pra** | **ACEI** | OR | 0.943 | 0.935 | 0.956 | 0.933 | 1.068 | 0.899 |
|  |  | CI | (0.875-1.016) | (0.857-1.020) | (0.839-1.088) | (0.860-1.013) | (0.844-1.351) | (0.659-1.226) |
|  |  | p | 0.120 | 0.131 | 0.494 | 0.098 | 0.583 | 0.501 |
|  |  |  |  |  |  |  |  |  |
| **Rosu** | **ACEI** | OR | 0.857 | 0.869 | 0.833 | 0.880 | 0.687 | 0.817 |
|  |  | CI | (0.784-0.937) | (0.782-0.967) | (0.703-0.987) | (0.796-0.973) | (0.491-0.961) | (0.634-1.053) |
|  |  | p | 0.001 | 0.010 | 0.035 | 0.013 | 0.028 | 0.119 |
|  |  |  |  |  |  |  |  |  |
| **Ator** | **ARB** | OR | 0.952 | 0.984 | 0.858 | 0.952 | 0.927 | 0.935 |
|  |  | CI | (0.896-1.010) | (0.920-1.053) | (0.756-0.973) | (0.888-1.020) | (0.739-1.162) | (0.772-1.132) |
|  |  | p | 0.103 | 0.644 | 0.017 | 0.164 | 0.509 | 0.488 |
|  |  |  |  |  |  |  |  |  |
| **Sim** | **ARB** | OR | 0.941 | 0.959 | 0.897 | 0.950 | 0.957 | 0.896 |
|  |  | CI | (0.895-0.990) | (0.907-1.014) | (0.813-0.989) | (0.899-1.003) | (0.819-1.117) | (0.740-1.084) |
|  |  | p | 0.019 | 0.145 | 0.029 | 0.065 | 0.576 | 0.258 |
|  |  |  |  |  |  |  |  |  |
| **Pra** | **ARB** | OR | 0.813 | 0.844 | 0.713 | 0.830 | 0.547 | 1.026 |
|  |  | CI | (0.739-0.895) | (0.760-0.939) | (0.571-0.890) | (0.742-0.929) | (0.355-0.842) | (0.767-1.372) |
|  |  | p | <0.001 | 0.002 | 0.003 | 0.001 | 0.006 | 0.865 |
|  |  |  |  |  |  |  |  |  |
| **Rosu** | **ARB** | OR | 0.833 | 0.866 | 0.738 | 0.777 | 1.101 | 0.826 |
|  |  | CI | (0.752-0.922) | (0.774-0.968) | (0.595-0.916) | (0.687-0.879) | (0.798-1.519) | (0.646-1.056) |
|  |  | p | <0.001 | 0.011 | 0.006 | <0.001 | 0.558 | 0.128 |
|  |  |  |  |  |  |  |  |  |
| **N** |  |  | 2,185,119 | 1,357,712 | 827,407 | 1,818,216 | 142,553 | 118,645 |
| Logistic regression results for AD incidence in sample of 2009-2014 Medicare person-years with 90 possession days and 2 claims of both an AHT and a statin in both years t-1 and t-2. AHTs are antihypertensive (AHT) prescription drugs (angiotensin converting enzyme inhibitors (ACEIs), angiotensin-II receptor blockers (ARBs), beta-blockers, calcium channel blockers, loop diuretics, and thiazide diuretics), and statins are atorvastatin, pravastatin, rosuvastatin, and simvastatin. Sample restricted to person-years with 3 years fee-for-service, 3 years Part D, age 67+, no deaths in the reference year (year t), no prior AD diagnoses, and no prior use of acetylcholinesterase inhibitors (AChEIs) or memantine. Controls are age, age squared, sex, education, income quartiles, statin use (t-1), years since hypertension and hyperlipidemic diagnoses, HCC comorbidity index, number of physician visits, and indicators for past diagnoses of diabetes, atrial fibrillation, acute myocardial infarction, and stroke. Standard errors are clustered at the county level. | | | | | | | | |
