# Supplementary material for: Association of combination statin and antihypertensive therapy with reduced Alzheimer’s disease and related dementia risk
Source: PLoS One. 2020 Mar 4;15(3):e0229541. doi: 10.1371/journal.pone.0229541 (PMC7055882; doi:10.1371/journal.pone.0229541)
Supplement: Attachment — Submitted filename: Response to reviewers - Statins+AHT - 20 01 16.docx [file pone.0229541.s005.docx]

We thank the reviewers for their important comments and suggestions, which are shown in italics below. The manuscript is much improved as a result of their feedback. The portions of the main text of the paper that were changed for the resubmission are shown in track changes in the file titled “Revised Manuscript with Track Changes.” Please see our specific responses to the reviewers’ comments below.

**Reviewer 1’s comments and suggestions: *Reviewer #1:*** *Thank you for the opportunity to review this manuscript. Overall, I was happy with the conduct of the study, the large sample size and robust analysis. The findings have high clinical interest as the problem of Alzheimer’s/related dementias is very prevalent, as is co-morbidity of hypertension and hypercholesterolemia in older people. Given that statins and antihypertensive drugs are widely used, the finding that certain commonly used combinations might offer greater protection against Alzheimer’s/related dementias than others is of great relevance.

There is room for improvement in this manuscript in two areas.*

We thank the reviewer for their support of this work, and for providing such detailed feedback. Please see below for our responses.

***A) Issues in the descriptions relating to the clinical pharmacology of the drugs studied***

***1) Line 295*** *– ‘The pharmacology and pharmacokinetics of rosuvastatin are distinct’. First, this is strangely worded as pharmacokinetics are an aspect of pharmacology – this would be better phrased as ‘the pharmacokinetics and pharmacodynamics …’. I would like to see this statement explained further – in what way is rosuvastatin pharmacologically different from the others? Does this extend beyond the pharmacokinetic issues discussed in the following lines to some unique pharmacodynamic/mechanistic property ? I would think that explaining these differences are central to the argument that there is a pharmacological justification underlying the findings.*

We thank the reviewer for their detailed attention to this wording, as well as the pharmacology content throughout this paper. We have adjusted the wording as recommended, added a citation that better supports this statement, and expanded our explanation of how these attributes may be related to our findings.

***2) Line 304.*** *The authors talk of the relative potency of the four statins differing from the observed relative benefits in protection against Alzheimer’s – which might mean the protective effect vs Alzheimer’s is mediated by a different mechanism from the LDL-lowering effect. But is potency the key measure here ? Is it not the product of potency and typical dosage – i.e. if drug A is 10 times as potent as drug B, but drug B is typically used at 20 times the dose of drug A, then we would expect drug B, not drug A to offer the most protection (assuming the anti-dementia effect is medicated through the same initial mechanism as the lipid lowering effect)? Presumably also, the statins all have similar efficacy in lipid lowering when used at their standard doses (which therefore offset the differences in potency) or else a literature would have emerged on certain molecules being associated with greater lipid-lowering efficacy than others. So essentially I am not convinced regarding lines the argument from line 304-306.*

The reviewer makes an excellent point. Indeed, we did not adjust our measurement of statin exposure for different doses, because we had assumed that statins are prescribed at the dose necessary to achieve a certain amount of LDL-reduction. We were therefore implicitly assuming that the doses prescribed of each molecule had an equivalent LDL-lowering effect. That sentence, as originally written in the manuscript, was imprecise, and we have removed it.

***3) Line 306-313*** *- The points relating to the CYP metabolizing enzymes are difficult to follow. There appear to be two points which need to be considered separately – i.e. a) there might be CYP-based pharmacokinetic interactions between certain statins and certain antihypertensive agents resulting in higher concentration/dose ratio of one or both drugs which might have an impact on the protective effect against ADRD and b) the impact of statins or antihypertensive drugs on activity of certain CYP enzymes might have a direct impact on risk of ADRD if the presence of the CYP enzyme is itself a risk factor or protective factor for ADRD. Point (a) is weakened by the fact that, according to Flockhart’s table, the statins and antihypertensives are substrates of certain CYP enzymes but are not strong inhibitors so there may often be sufficient metabolizing capacity that the co-prescription of two drugs metabolized by the same CYP may have no impact on their concentrations. As regards point (b) - the genetic variants in CYP expression (such as in CYP2C9 and CYP2C19 which metabolize rosuvastatin) and their ramifications for protection against dementia would already be inherent to the individual whether or not they ever took rosuvastatin (or any other substrate medication), and would only come in to play for the rosuvastatin if perhaps an individual was genetically already a low capacity metabolizer, or co-prescribed a 2C9/2C19 inhibitor, and the presence of rosuvastatin and the appropriate ARB could then inhibit what limited CYP2C9/CYP2C19 activity remained. All in all this is speculative and in my opinion the putative pharmacokinetic mechanisms have been given undue prominence (the CYP suggestions account for over half of the discussion of mechanisms). I would advise organizing this section more logically to cover the (a) and (b) points. The authors may wish to add a small table (or even a bulleted list) derived from Flockhart or similar sources, which allows the user to see which CYPs are involved in the metabolism of the four statins, and the main ARBs and ACE inhibitors and non-ARB antihypertensives, this would allow the potential interactions to be appraised quickly and easily and would demystify this section.*

We appreciate the suggestions regarding the organization of this section. Sorting the content into the pharmacokinetic interaction related mechanisms, and the direct relationship between CYP enzymes and ADRD risk factors makes sense, and we have reorganized this paragraph accordingly. We believe this is a concise and clear explanation of these possible mechanisms.

***4) Line 324*** *– I have some reservations with the statement that CYP3A4 is a ‘highly polymorphic enzyme’. It would be reasonable to say ‘polymorphic’. Evidence that this is of functional importance relative to other CYPs is limited. A review by Mizutani T(2003) Drug Metabolism reviews ‘PM Frequencies of Major CYPs in Asians and Caucasians’ pp99-106, stated a Poor Metabolizer frequency for CYP3A4 of 0% in Caucasians (table 2) and 1% in East Asians (table 1), the corresponding figures for CYP2D6 being 7% for in Caucasians and 0.8% in East Asians – while for CYP2C19 19% of East Asians are reported as being poor metabolizers. Looking at the paper the authors referenced (McGraw & Waller, ref 54) it appears that this paper lumps together CYP3A4 with CYP3A5 and describes more heterogeneity with CYP3A5 than CYP3A4. For CYP3A4 it mentions that a 3A4*1B allele is fairly common in African Americans and less so in Caucasians but also states that ‘the clinical impact of this polymorphism has not yet been identified’. There is however some potentially relevant evidence cited in this paper about a CYP3A4*22 allele (presumably associated with an extensive metabolizer phenotype) having impact on simvastatin metabolism. I think the case would be stronger here if you were to also cite and describe the original research relating to this allele (ref 63 Wang , Guo , Wrighton et al who refer to it as a ‘functional SNP located in intron 6’) as this paper provides direct evidence of a CYP3A4-related variant which impacts on statin metabolism.*

We have adjusted the wording in accordance with the reviewer recommendation. Indeed the evidence cited by McGraw and Waller (2012) is relevant to this point, and we have added additional content on the findings of Wang et al (2011). We thank the reviewer for these helpful suggestions.

***B) Issues in Sample Description and justification of representativeness***
 *Second, there are some shortcomings in the sample descriptions. In particular – and I think this is of particular relevance to readers not closely familiar with the healthcare system in the USA - the descriptions of the Medicare system (lines 153-158) are inadequate. The general reader needs to know how what is ‘Medicare’ and how is it funded – is this government funded care as is commonly found in Canada and the European Union, is it a wholly privately funded insurance system or some combination of the two? What proportion of US residents/citizens are covered by or registered for Medicare? Was the sample drawn from 20% of Medicare beneficiaries in one specific part of the USA or from 20% of the entire US population registered with correct form of Medicare, aged over 67 and meeting the remaining criteria? Crucially, there is no discussion of how representative the sample of individuals registered to receive Medicare included in the present study is of the general population in the USA. E.g. Are economically disadvantaged people and ethnic minorities under-represented or over-represented in the population registered to receive Medicare ? The representativeness of the sample must be discussed and if necessary listed as a limitation. I note that in line 271 the sample is described as ‘broadly representative’ but the authors have not given sufficient information to justify this assumption. Further the authors go on (in the discussion) to give further details about the Medicare-based sample using including concepts such as ‘FFS’ (‘fee for service’) and ‘part D’ which is described earlier (line 155) as “part D claims include key elements related to prescription drug events’ - I’m sorry but this is impenetrable to me and will similarly be impenetrable to some readers – these differing gradations of the Medicare system need to be explained in more generic terms for readers unfamiliar with your system. What (line 366) is the ramification for the respresentativeness of the sample of excluding people in FFS without part D or in Medicare advantage? These concepts must be defined in the methods section or it is impossible for the reader to judge.*

We thank the reviewer for pointing out these deficiencies in the manuscript. We acknowledge that contextual information on Medicare is important to understanding the study, and non-US readers would have difficulty understanding much of this terminology. Accordingly, we have updated the manuscript to include these details. The description of Medicare’s funding, coverage, and sampling has been added to the Data section of the manuscript, and now clarifies that our sample of fee-for-service enrollees is slightly less healthy than those in Medicare Advantage on average, but still comprise a majority of the older adults in the United States. The terminology precision has been improved throughout, including the last paragraph of the discussion where we discuss the implications of including only beneficiaries with traditional Medicare.

***C) Further Question***
 *Do the authors consider that there is a possibility of ‘confounding by indication’ – i.e. certain patient characteristics encouraging prescribers to prefer or avoid certain drugs in patients with attributes which might themselves be risk factors for dementia. One example might be using beta blockers preferentially as an antihypertensive drug of choice in patients exhibiting longstanding anxiety which could, through its associations both with depression and cardiovascular disease be associated with development of dementia. I am not suggesting that this is a sufficiently important issue that it demands that some mitigating methodology such as propensity-score matching should have been used, but I think the possibility of this occurring should be mentioned.*

Confounding by indication is often a major limitation of pharmacoepidemiologic studies, and the reviewer is justified in raising this concern. This issue was a major factor that we took into consideration when we were forming our study design. By restricting our sample to individuals using both a statin and an antihypertensive, and only making comparisons within that group, we are inherently restricting to a set of individuals with relatively similar underlying health and healthcare utilization tendencies. Nevertheless, the reviewer is correct that other characteristics of the individuals could affect both choice of statin/antihypertensive, and risk of ADRD. As such, we included an extensive set of covariates in our regressions that control for a wide variety of confounding factors: age, sex, education, income, years since hypertension and hyperlipidemic diagnoses, hierarchical condition category (HCC) comorbidity index, number of physician visits, and indicators for past diagnoses of diabetes, atrial fibrillation, acute myocardial infarction, and stroke. Of particular importance on this list are the HCC index, which captures overall health status. Additionally, number of physician visits proxies for underlying health conditions and an individual’s use of the health care system. Education and income proxy for socioeconomic factors that can sometimes sort people into particular pharmaceutical treatment options.

Despite this extensive set of controls, we are aware that unobserved characteristics of individuals could still be confounding our analyses. As such, we noted in our original submission that a limitation of our study is the possibility that unmeasured bias remains (second sentence of last paragraph of discussion). We have expanded on this limitation, to specifically note the possibility of confounding by indication.

***D) Minor points***

***1) In the Abstract*** *– ‘FINDINGS’ section, the phrase “Pravastatin and rosuvastatin combined… “ is ambiguous, would be better phrased as “Pravastatin OR rosuvastatin combined…”*

The reviewer makes a good point, and we have corrected this as recommended.

***2) In the Abstract*** *– ‘METHODS’ section – the last sentence is a little confusing and would be improved by changing the final ‘and’ to “vs.”*

We thank the reviewer for this suggestion, and have adjusted the paper accordingly.

***3) In the Abstract*** *– ‘CONCLUSION’ section – I suggest changing ‘… is less common than other statins’ to is ‘is less common than use of simvastatin or atorvastatin. This is because other statins exist which are not mentioned in this manuscript, e.g. fluvastatin and lovastatin. This also applies in line 374 (conclusion).*

The reviewer makes a good point, and we have corrected this as recommended in both the abstract and the conclusion.

***4) In the Introduction****, lines 121 – 123. I would suggest providing more information on the observational studies rather than merely referencing that they exist. Being picky, it is actually incorrect to say that they are all ‘more recent’ than the RCTs. But I believe the reader would be better served if there could be some greater detail and critique about these studies (at least those that the authors view as the most important), as understanding what they have found is essential to the context of the present study.*

We agree that this summary of the literature could be improved with more detail on the results of the existing observational studies. While the literature in the area is quite large, we have added summaries of the key observational studies to the introduction as recommended.

***5) Line 135*** *– in defining ‘other AHT drug classes’ only calcium channel blockers, beta blockers and thiazides are mentioned – note the bracket is an ‘i.e.’ (suggesting there should be a complete list) and not an ‘e.g.’. The obvious missed class is loop diuretics which as I understand are still commonly used and were included in this study. What also of other less common AHT classes – alpha blockers, mixed alpha/beta blockers (e.g. carvidelol), vasodilators, centrally acting alpha-2 agonists? Can the authors add a line (around line 175) in the methods to justify excluding these less commonly used drug classes?*

We thank the reviewer for pointing out this oversight. We have corrected it in the updated manuscript.

***6) Line 293*** *– can the authors clarify if the loss of patent protection for Crestor/Rosuvastatin described in 2016 was specific to the United States?*

Yes, that patent expiration was indeed specific to the United States. We have clarified this in the manuscript.

***7) Line 320*** *– I wonder if ‘CYP2CP’ is a typo – the referenced Flockhart table (ref 53) lists CYP2C9 and CYP2C19 as well as the less familiar CYP2C8, presumably this should be CYP2C9? Alternatively perhaps this is not a typo and the authors are using ‘CYP2CP’ as an umbrella term to refer to all the CYP2C enzymes - this would however be an unusual usage. Either way this needs clarification.*

The reviewer is correct – this was a typo, and it has now been corrected. We thank the reviewer for their careful eyes!

***8) Conclusion*** *– I would suggest adding a sentence to state that these findings, if replicated, suggest a possibility that certain antihypertensive-statin combinations might be recommended preferentially to older adults in the interest of reducing risk of ADRD.*

While we are cautious to advocate clinical implications from our study, we believe that the reviewer is correct to note that if these findings are confirmed, certain treatment combinations should be preferred for certain patients. We have added this to the conclusion of the manuscript, as recommended by the reviewer.

**Reviewer 2’s comments and suggestions:**
***Reviewer #2:*** *This is an exciting paper using large databases of Medicare beneficiaries to examine the combination of statin and antihypertensive therapy (AHT) and related dementia risk. This is an important topic, because a common recommendation for patients to prevent dementia is to take statins and AHTs, although real-world data with large enough sample size to assess the effects of individual statin/AHT combos is limited. I only have some major and minor comments:*

We thank the reviewer for their enthusiasm for this research. Please see below for our responses to specific comments.

***1.*** *Is the overall combo of statin (any) + AHT (any) has a beneficial association with dementia, relative to a non-AHT, non-statin comparator? This could be reported in the abstract - since a clinician reading this paper, may get a "blanket message" that any combo is good - whereas perhaps only certain combinations are helpful*

We thank the reviewer for this important question regarding the relationship between the combination of any statin and any antihypertensive and ADRD risk, which is related to the pre-existing research that helped motivate our study. Past research on these drugs, including our own studies, has found protective associations between each of those groups of drugs and ADRD risk. Our own work found antihypertensives to be differentially protective across class, *while controlling for the use of any statin*. Similarly, we found statins to have a protective association, while controlling for the presence of hypertension (which is a strong proxy for antihypertensive use in this Medicare Part D population). This tells us that any antihypertensive combined with any statin could be protective, but this is not something we examined in this study. As explained in our introduction, this is because we had reason to hypothesize that if we compared specific combinations, the association with ADRD risk might vary. This hypothesized variation in effects on ADRD risk across different types of statins and antihypertensives relates to possible clinical implications in this population with high prevalence of combination use, and therefore motivated our comparison. Our study did not examine any statin in combination with any antihypertensive, nor has any literature that we are aware of; we therefore cannot make any statements about the protectiveness of such combinations.

***2.*** *It's not clear in the abstract what your primary question is - whether (or not) any combo of statin(any)/AHT(any) will be associated with improved outcome (this is what seems to be); and if not - whether you are particularly interested in certain drug combinations (and which of those is your primary question)*

The reviewer makes a good point about the clarity of the main objective in the abstract. Accordingly, we have added a sentence to the background section of the abstract that explains this.

***3.*** *Even though this is a large set - it's not clear whether the sample size was necessarily large enough to test each of the combinations separately - If this is the case, it should be reported in the limitations section.*

We thank the reviewer for this inquiry. Table 1 depicts the sample size, and shows that each of our eight exposure groups has at least 60,000 observations, and five of them have over 100,000 observations. Our logistic regressions compare these eight groups to the group using a non-RAS AHT + any statin, which is composed of 630,777 observations. Even with our extensive set of control variables, we are able to identify significant associations in these primary analyses. Our smallest subgroup analysis is for Hispanic individuals, and this regression still has a sample of 106,019 observations – here, the eight exposure groups each include at least 3,137 observations, and the comparison group includes 25,736 individuals. We are confident that these sample sizes are large enough to detect differences in ADRD risk across the statin+AHT combinations.

***4.*** *Study Design - This is not clear - is this a cohort study (which it seems to be) or is it a nested case control design or other design? Also it is not clear how long the maximum follow up (seems to be 5 years, but can be made more explicit)? Did you only include people with 2 years of AHT/statin use? What happened to people with shorter durations of use - were they excluded completely (ideal) or put in the reference group?*

We thank the reviewer for this question, and apologize for the confusion. This is retrospective cohort design. In each year t, from 2009-2014, we examine the association between ADRD risk in year t, with statin+AHT use in years t-1 and t-2. For example, in 2011, the relevant drug use occurred in 2009 and 2010. We apply our full set of inclusion/exclusion criteria (explained in the Study sample section) to the observations that are included in the analysis in each year, from 2009-2014. Finally, these observations are pooled, so that the full analytic sample is a pooled set of 6 different 3 year exposure-outcome windows, for each of the 6 years 2009-2014. A single individual could contribute a data point to more than one year, as long as they met the inclusion/exclusion criteria in those years.

As explained in the Study sample section, part of the inclusion criteria for a single year (t) was that the individual must have used both a statin and an AHT in both year t-1 and year t-2. As explained in the section on Measuring statin and AHT exposure, users were defined as those with at least 90 days supply and at least two claims in both year t-1 and year t-2. Anyone who did not meet these criteria was excluded from the analysis.

We recognize that this design is complex, and have worked to improve the clarity with revisions to three sections: Study sample, Measuring statin and AHT exposure, and Study design.

***5.*** *Another important limitation of the study is that it used a prevalent user (of AHT/statin) cohort - as opposed to a new-user design (previously unexposed to AHT/statin), which weakens causality. This should be mentioned in the limitations*

The reviewer is correct that we only observe prevalent use of AHTs. We have acknowledged that this could lead to confounding of the analysis in the 5^th^ sentence of the last paragraph of the discussion (the limitations section), especially since we cannot observe drug utilization prior to the introduction of Medicare Part D (in 2006). As noted, we therefore included control variables for time since hyperlipidemia diagnosis and hypertension diagnosis in the analyses. These diagnoses are measured back to 1999, and proxy for treatment of those conditions. Nevertheless, this is indeed a limitation, and we have acknowledged it as such.

***Minor comment:
6.*** *Sometimes ORs are reported in three digits - OR=0.942 - the industry standard is sometimes 0.94 in such cases. The journal could let you know if they have a preference for how to report such statistcis.*

The journal has stated flexibility on this standard, and we believe that three digits is appropriate, as it is consistent with our previous studies on this topic.

***7.*** *Ideally a statistician reviewer from the journal should also double-check this paper.*

We agree that additional review from a statistician is an excellent way to ensure the validity of the methods and their reporting. While we are confident in the work we have done, we would happily invite such a review.
